# Effect of Micellar Aggregate on the Kinetics and Mechanism of the Reaction between Ethylene Glycol and Periodate

**DOI:** 10.1155/2014/680176

**Published:** 2014-10-29

**Authors:** Olaseni Segun Esan

**Affiliations:** Department of Chemical Sciences, Adekunle Ajasin University, PMB 001, Akungba-Akoko, Nigeria

## Abstract

The oxidation of ethylene glycol by periodate (IO_4_^−^) was studied in different micellar aggregates of cetyltrimethylammonium bromide (CTABr) and dodecylamine (DA) by means of UV/Vis spectroscopy. The observed constant *K*_*o*_ was obtained by monitoring the disappearing of ethylene glycol with time at a suitable wavelength under pseudofirst condition. Addition of CTABr and DA inhibits the reaction rate while the kinetic behavior was explained on the association of one of the reactants with the micelles leaving the other reactant in the bulk solution (pseudophase model).

## 1. Introduction

Micelles are ultramicroscopic units in colloids and possess all the physical properties of colloid [1]. They are formed as earlier indicated as a result of aggregation of three or more molecules of surfactant existing in a particular liquid medium in thermodynamically stable equilibrium that create highly anisotropic interfacial region lining the boundary formed by polar aqueous and nonpolar hydrocarbon regions, impacting new chemical and physical properties to the system [2–7]. Determination of reaction rates in micellar is usually based on the pseudophase mode [1], which treats aqueous, organic, and/or surfactant components of the solvent medium as constituting distinct phases in which reaction occurs and between which reagent and product are distributed in accordance with conventional laws of kinetic and mass transfer.

Many substrates have been oxidized using periodate as oxidant [8]. Periodate as an oxidant has been used severally in organic chemical reaction [9]. It has the greatest application in the field of alcohol and carbohydrate chemistry [10]. Under controlled conditions, periodate will selectively oxidize 1,2-diol, 1,2-amino alcohols, 1,2-hydroxyl aldehyde, and ketones and various other groupings [11]. Periodate oxidation has a lot of advantages which are responsible for its being widely studied. For example, it can be applied in aqueous solution over a wide range of pH. Ethylene glycol is an organic compound primarily used as a raw material in the manufacture of polyester fibers and fabric industry. A small percent is used in industrial application like antifreeze formulation and other industrial products. The kinetic oxidation of ethylene glycol by various oxidizing agents has been investigated and was found to involve two electron transfers through the formation of a negatively charged cyclic intermediate.

Kinetics and mechanism of the reaction between ethylene glycol and periodate in micellar system remain unexplored. In this work, I have explored the effect of cationic (cetyltrimethylammonium bromide, CTABr) and nonionic (dodecyl amine, DA) surfactant micelles on the kinetics and mechanism of the reaction between ethylene glycol and periodate.

## 2. Experiment

Cetyltrimethylammonium bromide (CTABr) from Fluka, dodecyl amine (DA) from Sigma, and sodium periodate from BDH (99% pure) were used without further purification. Ethylene glycol (BDH) was purified by simple distillation. The water used in the preparation of solution was doubly distilled.

### 2.1. Kinetic Measurements

Reaction kinetics were studied on the Perkin-Elmer UV/Vis spectrophotometer, Lambola E 2150, using a cell of path length 1 cm by recording the change in absorbance due to disappearance of periodate (at 225.4 mn) in a thermostated reaction cell. The concentration of ethylene glycol was kept in large excess over the concentration of periodate. The kinetics were studied by the integration method. The integrated first order equation is as follows:

(1) $$\begin{array}{c} \ln\left(A_{t}-A_{\infty }\right)=\ln\left(A_{0}-A_{\infty }\right)-k_{0}t, \end{array}$$

where *A*_0_, *A*_*t*_, and *A*_*∞*_ are the absorbance time zero, *t*, and infinity was fitted to the kinetic data by using algorithm to give the first order pseudoconstant *k*_0_ (*k*_0_ = observed rate constant). The value of the observed rate constant was reproducible within the experimental error (3%).

### 2.2. Critical Micelle Concentration (CMC) Determination

The conductivity measurement was performed with Jenway 4510 digital conductometer using a dip-type cell of constant 0.88 cm^−1^. All measurements were done in a jacketed vessel, maintained at desired temperature, with circulating water thermostat bath. The conductometric method was used to determine the CMC value of CTABr and DA solution at different experimental conditions: CTABr, and DA only, CTABr + IO_4_^−^, CTABr + EG, DA + IO_4_^−^, and DA + EG.

The CMC value was determined from the specific conductivity versus [CTABr] and [DA] in the presence and absence of IO_4_^−^ and EG. The CMC was determined from the break of specific conductance versus surfactant concentration plots [12]. The CMC was found to be 9.1 × 10^−4^, 1.54 × 10^−4^, 9.7 × 10^−4^, 1.01 × 10^−3^, 2.06 × 10^−4^, and 1.50 × 10^−4^ mol/dm^3^ for water + CTABr, water + DA, CTABr + IO_4_^−^ (1.051 × 10^−5^ mol/dm^3^), CTABr + EG (3.580 × 10^−3^ mol/dm^3^), DA + IO_4_^−^ (4.20 × 10^−5^ mol/dm^3^), and DA + EG (2.87 × 10^−3^ mol/dm^3^), respectively, at 25°C as shown in Tables 1(a) and 1(b).

## 3. Result and Discussion

### 3.1. Reaction in the Presence of CTABr

**Figure 1:**
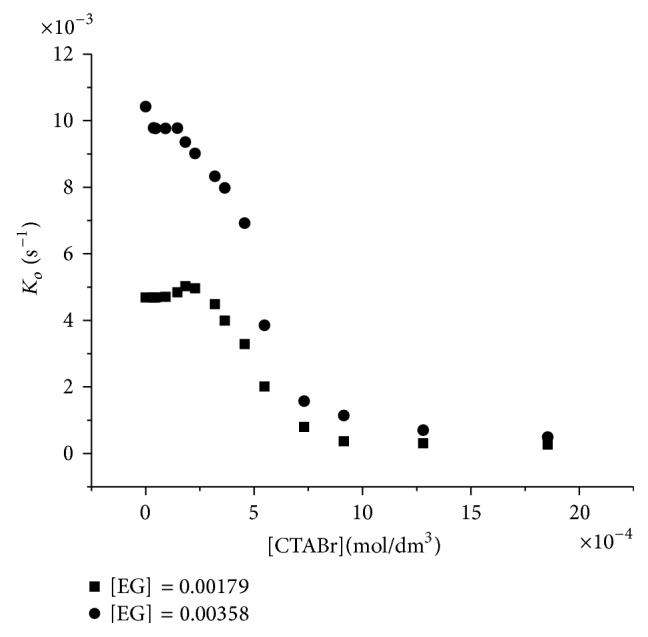
Influence of the concentration of CTABr upon the first order rate constant for the periodate oxidation of ethylene glycol (IO_4_^−^ = 4.266 × 10^−5^ mol/dm^3^) at 25°C.

The reaction was carried out in the presence of CTABr (0.000–2.743) × 10^−4^ mol dm^3^ and fixed concentration of EG and IO_4_^−^. Addition of CTABr results in partial increase in rate up to the concentration of 1.83 × 10^−4^ mol/dm^3^ after which inhibition predominate as shown in Figure 1.

The initial catalytic role of CTABr below 1.83 × 10^−4^ mol/dm^3^ can be explained on the fact that small aggregate of the CTABr exists below the CMC which interacts physically with the reactants forming active entities. Therefore, the catalytic role is due to the presence of premicelle and preponement of micellization by reactant; the two reactants are assumed to have penetrated the stern layer electrostatically [13]. Below 1.83 × 10^−3^ mol/dm^3^ of CTABr, inhibition occurs.

**Scheme 1:**
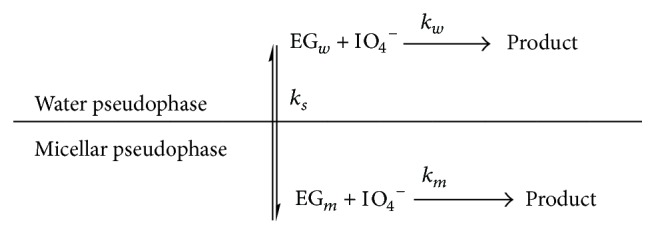


These could be interpreted using the kinetic model of the pseudophase proposed by Menger and Portnoy [14], which, taking the micelles as a pseudophase uniformly distributed in the aqueous phase, put forward a reaction scheme with a micelle-substrate equilibrium governed by an equilibrium constant *K*_*s*_ (Scheme 1). This scheme represents the micellized surfactant as *Dn* where [*Dn*] = [*D*] − CMC and [*D*] is the concentration of surfactant where *m* and *w* refer to the micellar and aqueous pseudophases, respectively. The scheme predicts a value for *k*_*ob*_ given by (2) which is the overall reaction rate. This value is equal to the rates at the micellar and aqueous pseudophases (Scheme 1)

(2) $$\begin{array}{c} k_{ob}=\frac{k_{w}+k_{s}k_{m}\left[Dn\right]}{1+k_{s}\left[Dn\right]}. \end{array}$$

The inhibition observed occurs because of the low concentration of IO_4_^−^ near the cationic surface causing the reactivity of the associated substrate to be much less than that of the substrate in the aqueous phase preventing the formation of [EG. IO_4_^−^] adduct.

If *k*_*m*_ = 0, IO_4_^−^ is completely excluded from the stern layer of the micelle and (2) becomes

(3) $$\begin{array}{c} k_{ob}=\frac{k_{w}}{1+k_{s}\left[Dn\right]}, \end{array}$$

where *K*_H_2_O_ represents the observed rate constant in the absence of surfactant.

Rearrange (3) to obtain

(4) $$\begin{array}{c} \frac{k_{w}-k_{ob}}{k_{ob}}=K\left(\left[D\right]-\text{CMC}\right). \end{array}$$

The CMC of CTABr and DA has been obtained as earlier discussed.

### 3.2. Reaction in the Presence of DA

**Figure 2:**
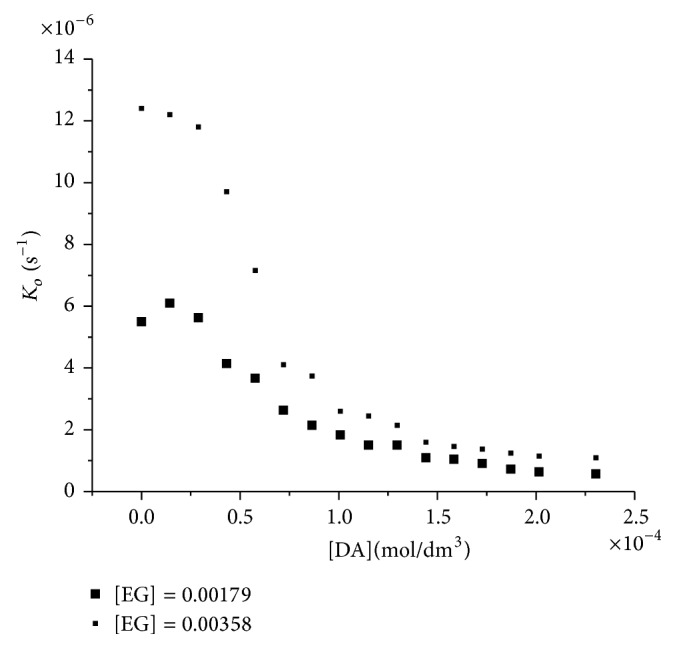
Influence of the concentration of DA upon the first order rate constant for the periodate oxidation of ethylene glycol (IO_4_^−^ = 4.266 × 10^−5^ mol/dm^3^) at 25°C.

The reaction was also studied in the presence of DA and the result (Figure 2) can be interpreted in the same way.

## 4. Conclusion

The result shows a biphasic pattern in both CTABr and DA. The reaction rate passes through a maximum as the surfactant concentration increases. This is due to two competing effects in the ion-exchange model. Added surfactant increases the relative concentration of EG and IO_4_^−^ in the stern layer which increases the reaction rate as shown by the ascending branch of the curve. As the concentration of CTABr and DA increases, the concentration of the reagent in the micellar pseudophase decreases and furthers the excess of unreactive IO_4_^−^ in the stern layer so that the reaction rate decreases. Finally, it can be concluded that, within the experimental range of studies, the pseudophase ion-exchange model has been found to be successful in explaining the result obtained in the kinetics and mechanism of the reaction between ethylene glycol and periodate in micellar system.

## Figures and Tables

**(a):** 

CTABr + substrate	CMC × 10^−4^ mol dm^−3^
CTABr	9.10
CTABr + IO_4_^−^	9.70
CTABr + EG	0.101

**(b):** 

DA + substrate	CMC × 10^−4^ mol dm^−3^
DA	1.54
DA + IO_4_^−^	2.86
DA + EG	1.50
